# Links Between Attention-Deficit/Hyperactivity Disorder Symptoms, Peer Relationships and Mental Health Outcomes in Western Australian Youth

**DOI:** 10.3390/children11111321

**Published:** 2024-10-30

**Authors:** Carolyn Maxwell, Stephen Houghton, Elaine Chapman

**Affiliations:** Graduate School of Education, The University of Western Australia, 35 Stirling Highway, Perth 6009, Australia; carolyn.maxwell@uwa.edu.au (C.M.); elaine.chapman@uwa.edu.au (E.C.)

**Keywords:** peer relationships, attention-deficit/hyperactivity, ADHD, mental health, adolescents

## Abstract

Peer relationships are critical in the development of positive mental health during childhood and adolescence. Attention-deficit/hyperactivity disorder (ADHD) symptoms can adversely affect the development of positive peer relationships, and thus, have significant long-term implications for mental health. This study explored the long-term relationships between ADHD symptoms, peer relationships and mental health in Western Australian adolescents. Participants were drawn from a sample of 1489 young people: *n* = 623 males with a mean age of 13.79 years (*SD* = 1.61) and *n* = 866 females, with a mean age of 14.29 years (*SD* = 1.51). Data were collected at three timepoints across three successive years, with different numbers of participants contributing data at each timepoint. Participants completed measures of ADHD symptoms and existing peer problems in the first year; five measures of peer relationships in the second year; and four measures of mental health in the third year. Multiple regression and path analyses were used to determine whether ADHD symptoms predicted scores on the peer relationship and mental health measures and whether peer relationships mediated links observed between ADHD symptoms and mental health. ADHD symptoms significantly predicted both long-term problematic peer relationships and long-term adverse mental health outcomes. Three peer relationship variables were significant mediators of relationships between ADHD symptoms and mental health: sense of belonging, friendships and perceived isolation. ADHD symptoms significantly predict poor peer relationships and adverse mental health outcomes in Western Australian youth, regardless of whether a formal diagnosis has been assigned. Early interventions for young people with ADHD symptoms are needed for such individuals to enjoy positive mental health in their adult years.

## 1. Introduction

Adolescence, generally defined as the period from 13 to 18 years of age, is a time of rapid and dramatic physical, emotional and social change. Alongside the hormonal and physical changes that occur during puberty, adolescence is marked by significant psychosocial developments in the cognitive, emotional and self-concept domains, exposing adolescents to increased risk with respect to mental health problems [1]. As a result, many mental health difficulties seen over one’s lifespan first appear during adolescence [2].

The mental health status of adolescents has significant impacts on how these individuals engage with various aspects of their lives. Mental disorders—along with substance use disorders—are the leading causes of disability in adolescents, accounting for a quarter of disability in young people worldwide [3]. The National Children’s Mental Health and Wellbeing Strategy [4] noted that, in 2015, “anxiety, depressive disorders and conduct disorders accounted for three of the five leading causes of disease burden for children aged 5–14 years” [4] (p. 17). Many mental health problems first seen in adolescence will also persist into adulthood [1]. Even those who no longer have a diagnosable disorder as adults have been found to function at a lower level throughout their adult years than those with no history of a mental disorder [4].

### 1.1. Importance of Healthy Peer Relationships for Positive Mental Health

Good peer relationships are essential for the healthy social development of children and adolescents [5,6], serving a protective function against the development of mental health problems. Conversely, negative peer relations have been found to have adverse effects on mental health, forecasting maladaptive social, emotional and behavioral developmental trajectories [7]. This is of particular significance during adolescence, as peer influences become increasingly important. If children and adolescents are unable to establish and maintain positive peer relationships or friendships, this can increase the risk of antisocial behaviors, substance abuse and psychological problems in later life [8].

Many young people will change schools as they enter adolescence and this can disrupt the existing friendships from their primary school, creating the need to establish new peer relationships. If children and adolescents are unable to establish and maintain positive peer relationships or friendships, these deficits can have serious consequences, including an increased risk of antisocial behavior, substance abuse and psychological problems in later life [8]. Adolescence is also the developmental period that carries the highest risk for loneliness [9]. Loneliness is strongly linked to a host of physiological and psychological health problems and is, in fact, a predictor of future depression, self-harm and suicidal ideation [9].

A range of factors that are critical in forming positive peer relationships in adolescence have been identified. Amongst these, social competence, which refers to an individual’s ability to adjust to different or variable contexts and to modify his or her behavior based on the demands of each situation, has been identified as crucial for forming positive peer relations. Young people with low social competence often fail to adapt their behavior effectively in different social contexts and are thus more likely to experience victimization (bullying) by peers [10]. Various mental health disorders place individuals at heightened risk of exhibiting social competence impairments during the adolescent years.

### 1.2. Attention-Deficit Symptomatology, Peer Relationships and Mental Health

Attention-deficit/hyperactivity disorder (ADHD) is the most common childhood neurodevelopmental disorder worldwide [11], affecting approximately 7.8% of Australian children and adolescents [12]. It is characterized by persistent, age-inappropriate inattention and/or hyperactivity–impulsivity that interferes with functioning across multiple settings, having effects in the domains of cognition, attention, behavior and emotion [13,14]. ADHD is characterized as predominantly inattentive, predominantly hyperactive–impulsive, or a combined presentation [13], depending on the dominant nature of the symptoms exhibited.

Young people with ADHD have been found to confront significant challenges in forming positive peer relationships [15,16]. Various social risk factors tend to be accentuated in young people with ADHD, which include:

(i) A lack of inhibition, which can lead to problems in co-operating or taking turns, as well as overbearing, intrusive or annoying behavior, which can be aversive to peers [16,17,18,19,20];
(ii) Socio-cognitive deficits, which can precipitate failures to adjust responses appropriately in different social contexts, difficulties in recognizing social cues and misunderstandings of peers’ intentions and behaviors [10,16];
(iii) Emotional dysregulation, which can prompt reactivity and frustration, having an adverse effect on behavior in social situations [17,21].

Peer rejection and victimization are two of the most serious relationship difficulties commonly experienced by adolescents with ADHD. More than half of children and young people with ADHD have been found to be rejected by their peers [22]. Children and adolescents with ADHD are not only at a higher risk of becoming victims of bullying by their peers [10,15,20], but are also more likely to act as perpetrators of bullying than those without ADHD [16,23,24]. For example, one study [24] indicated that 57.7% of 9- to 14-year-olds with ADHD reported having been victimized, bullying others, or both, as opposed to only 13.6% of those in a comparison group.

Young people with ADHD are documented to experience significant challenges with their mental health. The Raine ADHD Study [25] reported that children with ADHD “perform significantly worse at age 14 years on measures of depression” (p. 5) than those without the disorder. In fact, children with ADHD are five times more likely to suffer from depression and three times more likely to have anxiety, in comparison to their neurotypical peers [26]. Youth with ADHD also frequently have comorbid psychiatric disorders, the most common of these including oppositional defiant disorder (ODD), conduct disorder (CD), depression and anxiety [26].

In the Diagnostic and Statistical Manual of Mental Disorders, Fifth Edition (DSM-5), the notion of ‘presentations’ of ADHD reflects research evidence that ADHD is not a fixed disorder [14]. Rather, its expression is changeable across different stages of life and within varying contexts. The research evidence makes clear that adolescents’ ADHD symptoms are to a large part context-dependent [27], can fluctuate from day to day [28] and can vary depending on the context in which the adolescent is functioning [9]. Furlong and Chen [14] also note that ADHD symptoms may vary in their presentation given variations in context, such as school versus home. The consequences of particular behaviors will vary across these contexts, with some contexts being more tolerant. Furthermore, expectations imposed will change with age, such that behaviors accepted in younger children then become unacceptable by both peers and adults in adolescence [14].

Given this, an individual with ADHD may display varying degrees of hyperactivity–impulsivity or inattention and move forward as well as backward along this presentation continuum at different points in their lives. This can mean that some young people will have difficulties related to ADHD symptoms without attracting a formal diagnosis [29]. Various researchers [4,30] concur that even when the severity of some children’s mental disorder symptoms falls below formal diagnostic thresholds, these individuals may nevertheless suffer significant functional impairments.

These points underscore the need for an increased awareness of sub-clinical ADHD symptomatology and its implications for longer-term outcomes, including those associated with peer relationships and mental health. From this perspective, even when impairments are ‘milder’ on the presentation continuum and thus do not attract a formal ADHD diagnosis, adolescents exhibiting these symptoms are still worthy of concern due to the severe immediate and long-term consequences of such symptoms.

### 1.3. The Present Study

The research reported in this paper was designed to address two primary goals. The first was to evaluate the extent to which attention-deficit/hyperactivity disorder (ADHD) symptoms measured at the start of the present study (Timepoint 1, November 2018) predicted peer relationships in Western Australian adolescents over an extended time period (through to April/May 2019, Timepoint 2). The second goal was to determine whether mental health measures from a further data collection timepoint (Timepoint 3, July/August 2020) were predicted significantly by ADHD symptoms at Timepoint 1 and also, whether peer relationships, measured at Timepoint 2, were significant mediators of any relationships between ADHD symptoms at Timepoint 1 and mental health outcomes at Timepoint 3. The focus in this study was on internalizing measures of mental health. The specific research questions addressed in the present study were

1. Are young people with ADHD symptoms more likely to report known risk factors in terms of peer relationships (school belongingness, being a bully victim, bullying and poor or no friendships) than those without such symptoms over the longer term?
2. Are young people with ADHD symptoms more likely to fare poorly in the longer term in terms of their mental health, as measured by levels of general wellbeing, depression and worry—amount and frequency?
3. Are any observed relationships between ADHD symptoms and long-term mental health significantly mediated by the impact of ADHD on peer relationships?

## 2. Materials and Methods

This study was undertaken as part of a larger longitudinal project examining the trajectories of loneliness and mental health in adolescents [15]. Thus, the participants formed a community sample, in which no deliberate attempts were made to include certain proportions of young people with ADHD symptoms (formally diagnosed or otherwise). The data for the present study were collected at three timepoints: Timepoint 1—November 2018; Timepoint 2—April/May 2019; and Timepoint 3—July/August 2020 (please note that Timepoint 3 in the present study was Timepoint 4 in the broader study).

### 2.1. Participants

Data for Timepoint 1 were available for 1489 young people, *n* = 623 males with a mean age of 13.79 years (*SD* = 1.61) and *n* = 866 females, with a mean age of 14.29 years (*SD* = 1.51). These participants were enrolled across seven randomly selected schools in Perth, Western Australia (five state/government and two non-government). These schools represented a range of socioeconomic status areas as indicated by their Index of Community Socio-Educational Advantage (ICSEA). The ICSEA is set at an average of 1000 (*SD* = 100), with higher ICSEA values indicating higher levels of educational advantage for students attending the school [31]. The ICSEA values for schools in the present study ranged from 939 to 1191, thus representing a range around the average level.

To be included in the present study, participants needed to have complete data for Timepoint 1 and also, to have complete data for either the Timepoint 2 or the Timepoint 3 collection. Given the longitudinal nature of the research, the number of participants varied considerably across the timepoints, as shown in Table 1. The reasons for the non-completion of the Timepoint 2 and 3 measures were typically to do with factors such as school transfers and events that precluded participation in the data collection. Also shown in Table 1 are the mean scores for each of these groups on the hyperactive/inattentive subscale of the Strengths and Difficulties Questionnaire (Youth Self-Report Version). As indicated, only negligible differences were evident in the mean scores across these groups.

### 2.2. Instruments

The participants completed seven instruments across the three study timepoints.

#### 2.2.1. Timepoint 1: Measures of ADHD Symptoms and Existing Peer Problems

The Strengths and Difficulties Questionnaire, Youth Self-Report Version (SDQ) [32], a brief screening measure for adolescents aged from 11 to 17 years old, was used to assess ADHD symptoms and existing peer problems. This instrument comprises 25 items which focus on difficulties in the emotional, conduct, hyperactivity/inattention and peer relationship domains (five items each), with an additional prosocial behavior subscale (five items). Previous studies on the psychometric properties of the SDQ have indicated strong reliability and validity attributes for this instrument [33]. Two subscales from the SDQ were used in the present study. These were the hyperactive/inattentive (ADHD) subscale and the peer relationship problems subscale. Following the rescoring of reverse-worded items, scores for the five items within each of these were averaged to provide a total score for each subscale.

#### 2.2.2. Timepoint 2: Peer Relationship Measures

The 24-item Perth A-Loneness Scale (PALs) [34] was used to measure loneliness in the current study. The PALs uses a six-point scale that ranges from ‘Never’ (0) to ‘Always’ (5), with higher scores indicating higher levels of loneliness. Houghton and colleagues [15,34,35,36] have conducted numerous studies in which the PALs has been demonstrated to have strong psychometric properties and to comprise four correlated factors, which are presented as separate subscales in the measure. The two subscales used in the present study were friendship-related loneliness (e.g., having trustworthy friends) and isolation (e.g., having few friends). Following the reversals of relevant items, the item scores were averaged for each of these two subscales to produce a single score for the friendship-related loneliness and isolation factors, respectively.

Items from the School Connectedness subscale of the California Healthy Kids Survey (CHKS) were used to assess perceived school belongingness in the present study. The CHKS is part of the overall California School Climate, Health and Learning Survey (Cal-SCHLS) System. Resnick et al. [37] previously developed a measure for school belongingness and the four items in the secondary CHKS are modified versions of items from that measure. The CHKS has been found to be theoretically and psychometrically sound [38]. The items from the school belongness subscale used in the present study were “I feel close to people at/from this school”; “I am happy with/to be at this school”; “I feel like I am part of this school”; and “I feel safe in my school”. A total score for school belongingness was calculated by averaging scores from these four items.

Two subscales, based on questions originally used by Bonnano et al. [39], were used to assess the frequency with which the participants experienced bullying, either as a victim or as a perpetrator (four questions on each). The items in each subscale measured different forms of bullying (i.e., physical, verbal, social and cyber-bullying). Following Bonnano et al. [39], students’ reports of overall bullying and victimization were not used; only reports of bullying and victimization for each of the four specific types of bullying and victimization were incorporated in the analysis. Average scores were generated for each of these subscales using the mean frequency scores of the four relevant items (i.e., “How often have you been physically/verbally/socially/cyber bullied?” and “How often have you taken part in physical/verbal/social/cyber bullying?”).

#### 2.2.3. Timepoint 3: Mental Health Measures

The Short Warwick–Edinburgh Mental Wellbeing Scale (SWEMWBS), a brief version of the Warwick–Edinburgh Mental Wellbeing Scale (WEMWBS), was used to provide a general measure of wellbeing at Timepoint 3. The SWEMWBS uses 7 of the WEMWBS’s original 14 statements about thoughts and feelings. The seven statements are positively worded with five response categories, ranging from ‘none of the time’ to ‘all of the time’. The SWEMWBS has been validated for use in populations aged 15 to 21 years [40]. The scores used in the present study were the Rasch-based transformed scores from the SWEMWBS, as these transformed scores approximate an interval level scale [41].

The Children’s Depression Inventory, Self-Report (Short) version was used to assess symptoms of depression. The CDI is available both in its original 27-item version and in a briefer 12-item version (the CDI-2). The latter version typically takes between 5 and 15 min to complete. Each item in both forms of the CDI has three statements and the respondent is asked to select the one answer that best describes their feelings over the past two weeks. The CDI-2 has been reported to have excellent psychometric properties [42] and yields a total score that is very comparable to the one produced by the full-length version. The total CDI-2 raw score was used in the present study.

Two subscales from the Perth Adolescent Worry Scale (PAWS) [43], a brief, psychometrically sound measure of worry for use with adolescents, was used as an index of wellbeing in the present study. The PAWS comprises 12 items, 6 relating to peer relationships and 6 relating to academic success and the future. Psychometric evaluations have affirmed that this instrument possesses excellent psychometric properties in terms of its reliability, construct validity and criterion-related validity [43]. There are two primary components of the PAWS, which relate to the frequency and the amount of worry experienced by respondents. The average rating for each component was used in the present study.

### 2.3. Data Collection Procedures

Approval to conduct the broader longitudinal study was obtained from the University of Western Australia Human Ethics Committee (approval number 2023/ET000730) as well as the Western Australian Department of Education (approval number D18/0207029). Each of the participating school principals also provided their consent for the broader study to be conducted and informed consent and/or verbal assent were obtained from each of the individual study participants and their parents. Participants completed the surveys online during their normal lessons across four separate occasions, over a period of approximately 28 months. At each data collection point, the survey was made available for 30 days to allow all the students to access and complete the questionnaires.

Each participant was provided with a unique identification code for the entire study period. This permitted students to remain anonymous to the researchers and also ensured that the data could be linked precisely across the three study timepoints in the present study. To ensure rigorous implementation procedures, each school principal nominated one teacher who was responsible for ensuring that the broader study processes were carried out with fidelity in his/her school. Each of these teachers received thorough written instructions to ensure that the procedures were carried out in a standardized manner across the study schools.

### 2.4. Data Analysis Procedures

The data were analyzed using a combination of IBM SPSS V23 and LISREL V11. Prior to conducting any analyses, data screening tests for all the relevant assumptions of each intended statistical procedure were performed to ensure compliance with these underlying assumptions. All such analyses produced satisfactory results with respect to the distributional assumptions of regression analysis.

## 3. Results

Initially, all analyses were performed separately for males and females. However, in each case, it was found that the pattern of relationships that emerged was similar across the two subsamples. As a result, the two samples (males and females) were combined for the purposes of all subsequent analyses. The results are presented in this section in line with the three research questions posed.

### 3.1. Research Question 1: ADHD Symptomatology and Peer Relationships

The first research question posed for the present study was Are young people with ADHD symptoms more likely to report known risk factors in terms of peer relationships (school belongingness, bullying and poor or no friendships) than those without such symptoms? To address this question, controlling for age, four hierarchical Multiple Regression Analyses (MRAs) were performed with age and SDQ-ADHD scores from Timepoint 1 entered as independent variables (respectively) and the five peer relationship measures were completed at Timepoint 2 (the PALS—friendships factor; the PALS—isolation factor; being bullied; bullying others; and the CHKS school belonging subscale) entered as dependent variables.

Furthermore, to ensure that the ADHD scores made a *unique* contribution to predicting peer relationship scores over and above any peer problems that participants were already exhibiting at Timepoint 1, a second set of hierarchical MRAs was performed, adding scores from the SDQ–Peer Problems subscale prior to the SDQ-ADHD subscale scores. The latter analysis was used to provide a stringent test of the unique contribution made for prediction by the ADHD subscale scores; that is, whether ADHD symptoms predicted peer relationship issues over and above any peer problems already exhibited at Timepoint 1. Means, standard deviations and bivariate correlations for the male and female subsamples are shown in Table 2.

The outcomes of all the MRAs conducted are shown in Table 3. In Table 3, the dependent measures are shown in Column 1. Against each of these dependent measures are the results of two MRAs, one which comprises only age in years and SDQ-ADHD scores as predictors, and a second in which SDQ-ADHD scores are entered after both age in years and SDQ–Peer Problems scores are entered. The latter analysis was included to provide a stringent test of the unique contribution made by SDQ-ADHD scores to prediction.

As indicated by the products of the first MRAs reported for each dependent variable in Table 3 (i.e., before SDQ peer problems were incorporated as a predictor variable), ADHD symptoms, as measured by the SDQ ADHD subscale, made a significant contribution to predicting all five of the peer relationship variables at Timepoint 2, over and above any variance accounted for by age. The *R*^2^ for each of these ranged from 0.04 through to 0.10 (4–10% variance accounted for by ADHD symptomatology). Given the large number of factors that can affect peer relationships, it is significant that these percentages are accounted for by ADHD symptoms alone. Thus, even when a formal diagnosis has not been delivered, exhibiting elevated levels of ADHD symptoms poses a significant long-term risk factor for difficulties in peer relationships.

Further affirmation of the unique contribution to predicting peer problems made by ADHD symptomatology was evident in the second MRA conducted for each of the dependent measures, in which the SDQ—Peer Problem scores were entered prior to the ADHD symptom scores. Again, in each case, ADHD symptoms made a statistically significant unique contribution to predicting peer problems over and above age and any peer problems that already existed. This result affirmed the merit in screening for ADHD symptoms in addition to assessing existing peer problems, to predict problems that students may confront in the longer-term.

### 3.2. Research Question 2: ADHD Symptomatology and Long-Term Wellbeing

The second research question posed was Are young people with ADHD symptoms more likely to fare poorly in the longer term in terms of their mental health, as measured by levels of general wellbeing, depression and worry (amount and frequency)? Again, to address this question, controlling for age, hierarchical MRAs were performed with age and SDQ ADHD scores from Timepoint 1 as independent variables (respectively) and the four measures of mental health completed at Timepoint 3 (the SWEMWBS, the CDI-2, the PAWS worry frequency, and the PAWS worry amount items) entered as dependent variables. Descriptive statistics and bivariate correlations for all the variables in the MRAs are shown in Table 4, with the outcomes of the MRAs shown in Table 5.

As indicated in Table 4, ADHD symptoms made a significant contribution to predicting all four of the mental health subscale scores over and above the contribution made by age. The *R*^2^ for each of these was sizeable, ranging from 0.05 through to 0.13 (5–13% variance accounted for by ADHD symptoms). The largest *R*^2^ was seen in the prediction of CDI-2 scores. Given the extended time period between Timepoint 1 and Timepoint 3, this suggests that ADHD symptoms have an enduring effect on key mental health outcomes in young people, contributing in the longer term to some 13% of score variance in depression scores and 8% to overall wellbeing levels. This makes it clear that even when a formal diagnosis has not been delivered, exhibiting elevated levels of ADHD symptoms poses a significant risk to sound mental health and wellbeing in the longer term.

### 3.3. Research Question 3. Peer Relationships as Mediators of Links Between ADHD Symptomatology and Long-Term Wellbeing

The third research question posed was Are any observed relationships between ADHD symptoms and long-term mental health significantly mediated by peer relationships (school belongingness; bullying; and friendships)? The goal of this analysis was to identify which of the peer relationship variables was most important in mediating relationships between ADHD symptoms and long-term mental health variables, to assist practitioners in setting meaningful intervention targets to interrupt long-term negative effects of ADHD symptoms on mental health. To address this question, an effect decomposition via path analysis was performed, in which ADHD symptomatology was the sole exogenous variable. In the second panel of the path analysis were the peer relationship (hypothesized mediating) variables, while the third panel included the four Timepoint 3 mental health measures. Descriptive statistics and bivariate correlations for all the variables in the path analysis are shown in Table 6, with outcomes of the path analysis shown in Table 7.

**Figure 1:**
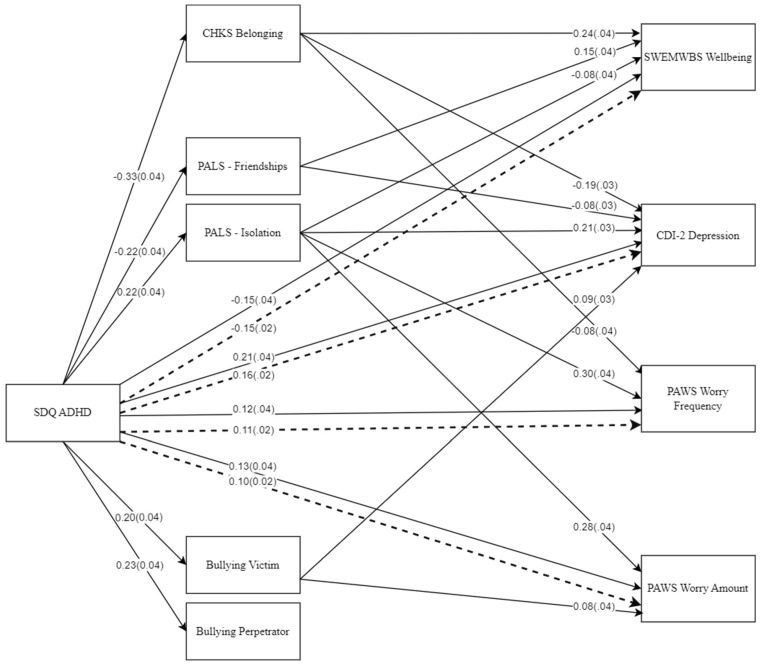
Significant effects in the model (direct effects solid arrows; indirect effects dashed arrows).

Figure 1 shows all the significant direct and indirect path coefficients generated within the model. As indicated, ADHD symptoms had significant direct and indirect effects on all four mental health variables. For wellbeing, the direct effect of ADHD symptoms was negative and significant, with the indirect effect also significant and negative. In contrast, the effect of ADHD symptoms on the remaining three variables was positive and significant for depression; worry frequency and worry amount. Interestingly, the primary indirect effects were not mediated significantly by the bullying variables, but were more mediated through the belonging, friendships and isolation peer relationship variables. Specifically, over half (50%) of the indirect effects of ADHD symptoms on the mental health outcome variables could be attributed to only one or two of the peer relationship variables—for wellbeing, to the belonging and friendship variables; for depression, to the belonging and isolation variables; and for worry frequency and amount, to the isolation variable.

## 4. Discussion

Outcomes of the present study affirmed that young people with ADHD symptoms are at an increased risk of exhibiting adverse mental health outcomes in the longer term, which can be attributed in part to the impact of ADHD symptoms on peer relationships. Given that participants were not necessarily formally diagnosed with ADHD, the results also underscored the need for the increased awareness of the potential implications of sub-clinical forms of ADHD for longer-term mental health. In other words, the results suggest that even in the absence of a formal ADHD diagnosis, adolescents exhibiting ADHD symptoms may still confront significant impairments in relation to these symptoms.

The results of the present study relating to Research Question 1 (Are young people with ADHD symptoms more likely to report known risk factors in terms of peer relationships than those without such symptoms over the longer term?) indicated that ADHD symptoms made a significant unique contribution to predicting all five of the peer relationship variables at Timepoint 1. The unique contribution made by ADHD symptomatology was further affirmed in the second set of MRAs, which incorporated, as predictors, the SDQ Peer Problems scores. ADHD symptoms continued to make a statistically significant unique contribution to predicting peer problems over and above that predicted both by age and by peer problems, affirming that even in the absence of a formal diagnosis, exhibiting elevated levels of ADHD symptoms poses a significant long-term risk factor for peer relationship difficulties.

In terms of Research Question 2 (Are young people with ADHD symptoms more likely to fare poorly in the longer term in terms of their mental health, as measured by general wellbeing, depression, worry—frequency and amount?), ADHD symptoms made a significant unique contribution to predicting all four of the mental health and wellbeing subscale scores over and above the predictive contribution made by age. The effect sizes were substantial, suggesting that between 5 and 13% of the total variance across the four subscales could be accounted for by ADHD symptoms. Considering the extended time period between Timepoint 1 and Timepoint 3, this result affirms that ADHD symptomatology is likely to have an enduring effect on key mental health outcomes in young people, contributing in the longer term to some 13% of score variance in depression scores and 8% to overall wellbeing levels. This makes clear that even when a formal diagnosis has not been delivered, exhibiting elevated levels of ADHD symptoms poses a significant long-term risk factor for mental health and wellbeing.

With respect to Research Question 3 (Are any observed relationships between ADHD symptoms and long-term mental health significantly mediated by peer relationships?), the focus was on identifying which of the peer relationship variables appeared to be most important in mediating the relationships between ADHD symptoms and the mental health variables. The path analysis performed to address this question indicated that ADHD symptoms had significant direct and indirect effects on all four of the mental health variables. For wellbeing, the direct and indirect effects of ADHD symptoms were negative and significant, while for depression, worry frequency and worry amount, the direct and indirect effects were all significant, but positive. The primary mediators associated with the significant indirect effects were the belonging, friendships and isolation variables. This highlights both the importance of recognizing long-term relationships between ADHD symptoms and mental health and also, the possibility of intervening upon these relationships, via the latter peer relationship variables.

While no previous studies could be located by the authors that addressed the specific questions addressed in the present study, the pattern of results aligns broadly with the previous literature in the field. This previous literature suggests that around half of all young people with mental disorders continue to have difficulties in adulthood [4]. The same body of literature has indicated that even those who go on to no longer have a diagnosable disorder as adults have a reduced chance of functioning effectively in comparison with those with no history of a mental disorder [4]. Previous longitudinal findings, which reported on the longer-term trajectories for mental health in young people, have affirmed adolescence as a period in which individuals are at particular risk of experiencing loneliness [34]. Loneliness, in turn, is a significant predictor of future depression, self-harm and suicidal ideation [34].

These results also align with previous findings related to the critical role played by effective social interaction skills in long-term mental health outcomes [15]. With specific reference to young people with ADHD, Lawrence et al.’s report on the Young Minds Matter survey indicated that some 60% of children and adolescents (4–17-year-olds) with ADHD reported problems with peer friendships [11]. In particular, 24.9% of those surveyed reported these problems to be ‘mild’ in severity, while a further 23.6% reported these problems to be ‘moderate’. Most alarmingly, 10.6% of children and adolescents surveyed reported having ‘severe’ problems in the area of peer relationships and with respect to friendships.

The relatively minimal contribution to mediation made by the two bullying variables was somewhat surprising. This could be attributed to the fact that schools in Australia, including Western Australia, have accelerated their efforts to address problems with bullying over the last decade. For example, the Western Australian Department of Education now requires every public school to have an anti-bullying plan and steps in place to deal with all forms of bullying. Schools, parents and children can also access a range of resources through the national Bullying No Way website [44], which is underpinned by significant federal and state funding. Bullying, therefore, may not have the same degree of impact on young people in Western Australia that it has had historically. In some respects, this was a positive finding of the present study.

Some cautionary elements are worth mentioning. There is a need for awareness that the varying reading ages of students could have impacted the comprehension of items in this self-report version of the SDQ. In a study of this nature, it was not possible to assess their individual reading ages versus their ages in years and to evaluate their comprehension of the questions asked of them. Also, the data were collected shortly before and then during the later stages of the COVID-19 pandemic. Thus, any effects on the participants’ mental health as a result of the pandemic were not able to be isolated and adjusted for.

With respect to some of the additional challenges that are likely to be confronted by students with ADHD symptoms in schools, Owens et al. [45] indicated that peer dynamics are clearly related to academic performance. Specifically, positive peer dynamics create a social context in the classroom that may foster the growth of academic ‘enablers’. Being poorly regarded by classroom peers may thus interfere with the development of important academic enablers in young people with ADHD symptoms. Therefore, developing classroom strategies that operate to support the development of positive peer relations, such as co-operative groupwork approaches and collaborative projects, may carry significant benefits for students with ADHD symptoms. The added advantage of such approaches is that these represent sustainable strategies that are more likely to produce the kind of durable behavior change that is needed to address peer problems definitively.

There would also be merit to screening for ADHD symptoms in addition to existing peer relationship problems as a long-term predictor of challenges. As noted, the primary mediators of the significant indirect effects seen in the present study were the belonging, friendships and isolation variables. This highlights both the importance of recognizing long-term relationships between ADHD symptoms and mental health and also, the need to isolate key mechanisms by which these relationships operate. Knowledge of these mechanisms would better equip schools to recognize *how* the adverse mental health outcomes of such students are developing over time and thus, to interrupt these relationships by focusing on what matters in terms of longer-term mental health outcomes.

Future research could explore in more depth the importance of friendship *quality* in mediating the relationships between ADHD and long-term mental health outcomes. Powell et al. [46] found that ADHD and symptoms of depression “were associated directly and indirectly via friendship quality, both for best friend and top three friends” (p. 1035) [46]. Thus, there is evidence that close, reciprocal friendships and a strong sense of belonging are protective factors against mental health problems. Also, given the evidence that children with ADHD symptoms have few close friends, with some reporting no reciprocated friendships at all [31,47], delving further into the importance of friendship quality and how this can be fostered in children with ADHD symptomatology may be a fruitful direction for future research.

## Figures and Tables

**Table 1:** Numbers of complete data sets for different timepoints in this study.

Completed Measures	No.	Mean SDQ ADHD Score (*SD*)	Male		Female	
			No.	Mean Age (*SD*)	No.	Mean Age (*SD*)
Any timepoint	1489	2.04 (0.47)	623	13.79 (1.61)	866	14.29 (1.51)
Timepoint 1 only	248	1.95 (0.48)	120	12.67 (1.74)	128	12.92 (1.83)
Timepoints 1 and 2 only	558	2.06 (0.50)	203	13.88 (1.60)	352	14.55 (1.45)
Timepoints 1 and 3 only	47	1.90 (0.49)	25	14.52 (1.50)	22	14.68 (1.25)
Timepoints 1, 2 and 3	636	1.95 (0.48)	272	14.15 (1.33)	364	14.49 (1.56)

**Table 2:** Descriptives and bivariate correlations for variables at Timepoints 1 and 2 (*n* = 1194).

Measure	Mean (*SD*)	Bivariate Correlations							
		1	2	3	4	5	6	7	8
1. Age in Years	14.32 (1.39)	--	0.05 *	−0.06 *	−0.06 *	0.05 *	−0.11 **	−0.02	−0.10 **
2. SDQ—ADHD	1.92 (0.48)		--	0.29 **	−0.22 **	0.23 **	0.20 **	0.21 **	−0.32 **
3. SDQ—Peer Problems	1.46 (0.39)			--	−0.45 **	0.44 **	0.36 **	0.14 **	−0.43 **
4. PALS—Friendships factor	27.42 (6.47)				--	−0.69 **	−0.41 **	−0.15 **	0.60 **
5. PALS—Isolation factor	10.89 (5.17)					--	0.40 **	0.12 **	−0.54 **
6. Bully Victim	6.25 (2.84)						--	0.40 **	−0.40 **
7. Bully Perpetrator	4.91 (1.79)							--	−0.17 **
8. CHKS Belonging	12.11 (2.62)								--

* Significant at 0.05 level; ** Significant at 0.01 level.

**Table 3:** MRA outcomes for Timepoint 2 peer relationship variables.

Dependent Measure	Predictor Entered	Model Statistics					Change Statistics				
		β	*R*	*R* ^2^	*R* ^2^_Adjusted_	*SE*	*R* ^2^_Change_	*F*_Change_	*df*1	*df*2	Sig.
PALS Friendships	Age in years	−0.05	0.06	0.00	0.00	60.46	0.00	4.92	1	1192	0.03
	SDQ-ADHD	−0.22	0.23	0.05	0.05	60.30	0.05	60.10	1	1191	<0.01
	Age in years	0.07	0.06	0.00	0.00	60.46	0.00	4.92	1	1192	0.03
	SDQ–Peer Problems	0.41	0.46	0.21	0.21	50.76	0.21	309.04	1	1191	<0.01
	SDQ-ADHD	0.10	0.47	0.22	0.22	50.73	0.01	11.82	1	1190	<0.01
PALS Isolation	Age in years	0.04	0.05	0.00	0.00	50.17	0.00	2.91	1	1192	0.09
	SDQ-ADHD	0.23	0.23	0.05	0.05	50.04	0.05	63.66	1	1191	<0.01
	Age in years	0.07	0.05	0.00	0.00	50.17	0.00	2.91	1	1192	0.09
	SDQ–Peer Problems	0.41	0.44	0.20	0.20	40.64	0.19	286.72	1	1191	<0.01
	SDQ-ADHD	0.10	0.45	0.21	0.20	40.61	0.01	14.62	1	1190	<0.01
Bullying Victim	Age in years	−0.12	0.11	0.01	0.01	20.83	0.01	13.46	1	1192	<0.01
	SDQ-ADHD	0.21	0.24	0.06	0.05	20.76	0.04	55.69	1	1191	<0.01
	Age in years	−0.09	0.11	0.01	0.01	20.83	0.01	13.46	1	1192	<0.01
	SDQ–Peer Problems	0.32	0.37	0.13	0.13	20.65	0.12	169.15	1	1191	<0.01
	SDQ-ADHD	0.12	0.38	0.15	0.14	20.63	0.01	17.19	1	1190	<0.01
Bullying Perpetrator	Age in years	−0.03	0.02	0.00	0.00	10.79	0.00	0.44	1	1192	0.51
	SDQ-ADHD	0.21	0.21	0.04	0.04	10.75	0.04	53.31	1	1191	<0.01
	Age in years	−0.02	0.02	0.00	0.00	10.79	0.00	0.44	1	1192	0.51
	SDQ—Peer Problems	0.08	0.14	0.02	0.02	10.77	0.02	22.17	1	1191	<0.01
	SDQ-ADHD	0.18	0.22	0.05	0.05	10.74	0.03	38.19	1	1190	<0.01
CHKS Belonging	Age in years	−0.08	0.10	0.01	0.01	20.60	0.01	12.14	1	1192	<0.01
	SDQ-ADHD	−0.32	0.33	0.11	0.11	20.47	0.10	135.06	1	1191	<0.01
	Age in years	−0.11	0.10	0.01	0.01	20.60	0.01	12.14	1	1192	<0.01
	SDQ—Peer Problems	−0.37	0.45	0.20	0.20	20.34	0.19	280.77	1	1191	<0.01
	SDQ-ADHD	−0.21	0.49	0.24	0.24	20.29	0.04	60.83	1	1190	<0.01

**Table 4:** Descriptives and bivariate correlations for variables at Timepoints 1 and 3 (*n* = 683).

Measure	Mean (*SD*)	Bivariate Correlations					
		1	2	3	4	5	6
1. Age in years	14.36 (1.25)	--	0.07 *	−0.13 *	0.14 *	0.10 *	0.12 *
2. SDQ—ADHD	1.91 (0.49)		--	−0.29 *	0.36 *	0.23 *	0.23 *
3. SWEMWBS Overall Wellbeing	2.74 (0.62)			--	−0.79 *	−0.43 *	−0.43 *
4. CDI−2 Depression	6.45 (4.78)				--	0.58 *	0.55 *
5. PAWS Worry Frequency	2.15 (0.57)					--	0.81 **
6. PAWS Worry Amount	2.22 (0.59)						--

* Significant at 0.05 level; ** Significant at 0.01 level.

**Table 5:** MRA outcomes for Timepoint 3 mental health variables.

Dependent Measure	Predictor Entered	Model Statistics					Change Statistics				
		β	*R*	*R* ^2^	*R* ^2^_Adjusted_	*SE*	*R* ^2^_Change_	*F*_Change_	*df*1	*df*2	Sig.
SWEMWBS Overall Wellbeing	Age in years	−0.11	0.13	0.02	0.02	0.62	0.02	11.61	1	681	<0.01
	SDQ-ADHD	−0.28	0.31	0.09	0.09	0.59	0.08	58.36	1	680	<0.01
CDI-2 Depression	Age in years	0.12	0.14	0.02	0.02	40.74	0.02	13.31	1	681	<0.01
	SDQ-ADHD	0.36	0.38	0.15	0.14	40.43	0.13	99.91	1	680	<0.01
PAWS Worry Frequency	Age in years	0.08	0.10	0.01	0.01	0.57	0.01	6.68	1	681	0.01
	SDQ-ADHD	0.22	0.24	0.06	0.06	0.56	0.05	34.65	1	680	<0.01
PAWS Worry Amount	Age in years	0.10	0.12	0.01	0.01	0.59	0.01	9.52	1	681	<0.01
	SDQ-ADHD	0.22	0.25	0.06	0.06	0.57	0.05	36.09	1	680	<0.01

**Table 6:** Descriptives and bivariate correlations for variables across Timepoints 1–3 (*n* = 636).

Measure	Mean (*SD*)	Bivariate Correlations									
		1	2	3	4	5	6	7	8	9	10
1. SDQ—ADHD	1.90 (0.49)	--	−0.22 **	0.22 **	0.20 **	0.23 **	−0.33 **	−0.29 **	0.36 **	0.23 **	0.23 **
2. PALS—Friendships	27.73 (6.25)		--	−0.71 **	−0.41 **	−0.18 **	0.57 **	0.40 **	−0.43 **	−0.28 **	−0.28 **
3. PALS—Isolation	10.43 (4.80)			--	0.42 **	0.13 **	−0.53 **	−0.37 **	0.46 **	0.37 **	0.37 **
4. Bullying Victim	6.27 (2.75)				--	0.40 **	−0.37 **	−0.27 **	0.34 **	0.25 **	0.26 **
5. Bullying Perpetrator	4.93 (1.85)					--	−0.23 **	−0.17 **	0.21 **	0.16 **	0.16 **
6. CHKS Belonging	12.33 (2.45)						--	0.44 **	−0.46 **	−0.29 **	−0.27 **
7. SWEMWBS Wellbeing	2.76 (0.61)							--	−0.79 **	−0.43 **	−0.43 **
8. CDI−2 Depression	6.37 (4.72)								--	0.57 **	0.54 **
9. PAWS Worry Frequency	2.15 (0.57)									--	0.80 **
10. PAWS Worry Amount	2.22 (0.59)										--

** Significant at 0.01 level.

**Table 7:** Direct and indirect effects from the path analysis.

Endogenous Variable	*R* ^2^	Predictor Variable					
		SDQ ADHD	Belonging	Friendships	Isolation	Bullying Victim	Bullying Perpetrator
PALS—Friendships	0.05	DE = −0.22 (0.04) *	--	--	--	--	--
PALS—Isolation	0.05	DE = +0.22 (0.04) *	--	--	--	--	--
Bullying Victim	0.04	DE = +0.20 (0.04) *	--	--	--	--	--
Bullying Perpetrator	0.05	DE = +0.23 (0.04) *	--	--	--	--	--
CHKS Belonging	0.11	DE = −0.33 (0.04) *	--	--	--	--	--
SWEMWBS Wellbeing	0.18	DE = −0.15 (0.04) * IE = −0.15 (0.02) *	DE = +0.24 (0.04) *	DE = +0.15 (0.04) *	DE = −0.08 (0.04) *	DE = −0.04 (0.03)	DE = −0.02 (0.04)
CDI−2 Depression	0.25	DE = +0.21 (0.04) * IE = +0.16 (0.02) *	DE = −0.19 (0.03) *	DE = −0.08 (0.03) *	DE = +0.21 (0.03) *	DE = +0.09 (0.03) *	DE = 0.05 (0.03)
PAWS Worry Frequency	0.16	DE = +0.12 (0.04) * IE = +0.11 (0.02) *	DE = −0.08 (0.04) *	DE = +0.04 (0.04)	DE = +0.30 (0.04) *	DE = +0.07 (0.04)	DE = +0.05 (0.04)
PAWS Worry Amount	0.14	DE = +0.13 (0.04) * IE = +0.10 (0.02) *	DE = −0.05 (0.04)	DE = +0.02 (0.04)	DE = +0.28 (0.04) *	DE = +0.08 (0.04) *	DE = +0.05 (0.04)

* Significant at 0.05 level.

## Data Availability

The data presented in this study are available on request from the corresponding author. The data are not publicly available due to ethical issues.
